# Atom’s Dynamics and Crystal Structure: An Ordinal Pattern Method

**DOI:** 10.1021/acs.jpca.4c06151

**Published:** 2025-01-17

**Authors:** Rafał Abram, Roman Nowak, Dariusz Chrobak

**Affiliations:** † Nordic Hysitron Laboratory, School of Chemical Engineering, Aalto University, Aalto 00076, Finland; ‡ Institute of Scientific and Industrial Research (SANKEN), Osaka University, Mihogaoka 8-1, Ibaraki, Osaka 567-0047, Japan; § Institute of Materials Engineering, University of Silesia in Katowice, 75 Pułku Piechoty 1A, 41-500 Chorzów, Poland

## Abstract

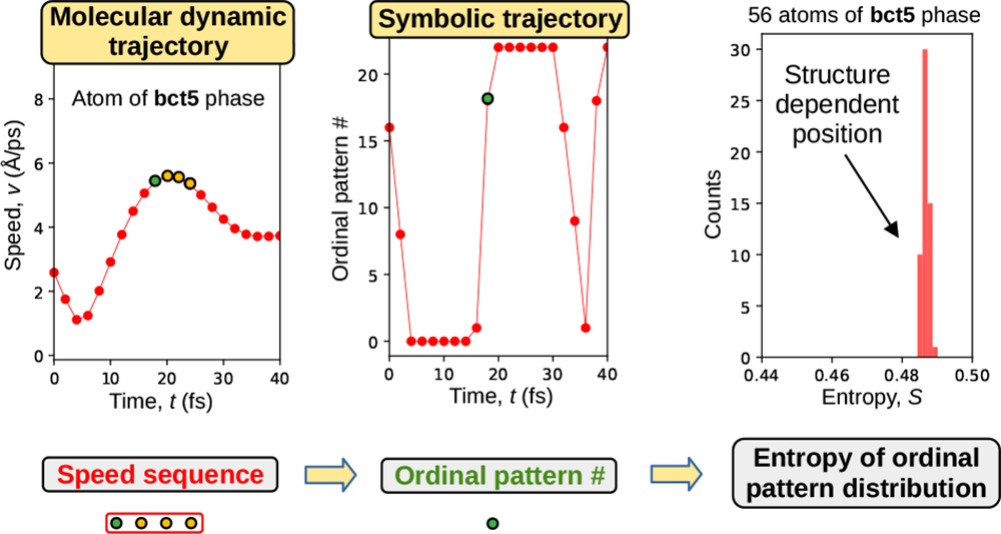

The ubiquitous nature of thermal fluctuations poses a limitation on the identification of crystal structures. However, the trajectory of an atom carries a fingerprint of its surroundings. This rationalizes the search for a method that can determine the local atomic configuration via the analysis of the movement of an individual atom. Here, we report, while using molecular modeling, how a statistical analysis of a single-atom speed trajectory, represented by ordinal patterns, distinguishes between actual crystal structures. Using the Shannon entropy of ordinal patterns enabled discernment of the studied high-pressure silicon phases. Identification of the atoms occupying the 2(c) and 6(f) Wyckoff positions of the r8 crystal revealed an increase in the developed method’s accuracy with trajectory length. The proposed concept of studying the structure of crystals offers new opportunities in solid–solid phase transformation studies.

## Introduction

Currently, numerical and experimental methods^1−5^ provide valuable structural data necessary to understand the properties of materials. The search for new algorithms for the determination of a material structure is ongoing, leading to exciting concepts, such as chaotic crystallography proposed by Varn et al.^6^ Regardless of the method employed, the accuracy of the crystal structure identification declines due to thermal fluctuations and imperfections in the crystal lattice. To overcome these obstacles, advanced machine learning techniques are currently being examined. However, despite many reported advancements proving the usefulness of this approach, it is computationally complex.^5,7−10^

This work presents a method for classifying atoms of different crystal structures. In the frame of molecular dynamics simulations, we described a single atom’s speed (the length of the velocity vector) trajectory in terms of ordinal pattern probability distribution. Then, we used Shannon entropy (simply entropy) to distinguish the local atomic arrangement. The selection of the atom’s speed did not limit the general nature of our approach, allowing the replacement of this parameter with other atom trajectory characteristics.

To verify our idea, we chose silicon because of its enduring presence in the scientific debate^11−15^ and, second, due to the difficulty in distinguishing of certain silicon high-pressure phases. In particular, there is a dilemma whether the appearance of the bct5 structure precedes the formation of the β-tin (Si–II) one during the indentation-induced transformation from cubic diamond (cd, Si–I) phase or is only a severely deformed original cubic lattice.^16,17^ Moreover, Gerbig et al.^17,18^ reported difficulties related to the analysis of Raman spectra of the metastable bc8 (Si–III) and r8 (Si-XII) phases, even though they are characterized by different space groups (*Ia*3̅ and *R*3̅, respectively) and Wyckoff positions: one 16(c) for bc8 and two 2(c) and 6(f) for r8 structure.^19^ We frequently referred to the crystallographic description of silicon structures by Mujica et al.^20^

## Methods

### Molecular Dynamics

The molecular dynamics simulations were performed with the LAMMPS code.^21^ To accomplish this, two interatomic potentials of silicon were selected, namely, the one proposed by Kumagai et al.^22^ due to its computational efficiency and the Spectral Neighbor Analysis Potential (SNAP)^23^ parametrized for silicon by Zuo et al.,^24^ portrayed as computationally expensive but accurate in modeling of silicon structures. The Kumagai potential was used for most of the phases studied in this work: cd, bct5, β-tin, and bc8, while we employed the Zou potential to test our statistical method on the bc8 and r8 phases. Since the temperature and pressure affect the local atomic arrangement, we conducted computational experiments at phase equilibrium points so that the atomic configuration is the only contrast between silicon structures. The atomic trajectory has been characterized by the entropy of ordinal pattern probability distribution^25−27^—the approach popular in various areas of science and technology.^28−30^

We combined the crystalline silicon phases in pairs. Each phase of a given pair was modeled separately at the same temperature and pressure, consistent with the applied interaction potential. To find the equilibrium pressure for the cd/bct5, bct5/β-tin, and β-tin/bc8 pairs of Si phases, we used the zero-temperature approximation of the enthalpy pressure dependence: *H*(*p*) = *E* + *pV*. The calculations consisted of stepwise changes of the pressure *p* and subsequent minimization of the potential energy to get the system energy *E* and the system volume *V*. For example, the solution to the equation *H*_cd_(*p*) = *H*_bct5_(*p*) gave the equilibrium pressure for the cd and bct5 phases. The results are shown in Table 1 and Figure S1 in the Supporting Information. The differences between the equilibrium pressures modeled with the Kumagai potential and the literature data were a consequence of applying the different interaction models or experimental conditions.

**Table 1:** Equilibrium Pressures *p* of Selected Si Phases Modeled by Means of the Kumagai Potential^22^

equilibrium	*p* (GPa)	references
cd/β-tin	12.2	6.34–16.5,^31^ 11.7^20^
cd/bct5	10.2	12.6^32^
bct5/β-tin	15.8	
bc8/β-tin	7.2	6.9,^33^ 7.4^19^

After determination of the phase equilibrium pressures, we generated atomic trajectories corresponding to the temperature of 300 K using supercells (containing ∼8000 atoms) of the cd, bct5, β-tin, and bc8 phases at the pressures indicated in Table 1. An isothermal–isobaric ensemble and Nose–Hoover dynamics^34−36^ were used with a time step of 2 fs. The simulations were preceded by energy minimization using the conjugate gradient algorithm and subsequent equilibration for 200 ps. Then, 2 ns (10^6^ time steps) long atomic trajectories of a group of atoms, selected from each of the cd, bct5, β-tin, bc8, and r8 phases, were recorded. The atoms were chosen so that the motion of one atom does not directly affect the motion of an adjacent atom; they are therefore not nearest neighbors. Each atom’s trajectory (time series) contained information regarding its coordinates (*x*, *y*, *z*) and velocities (*v*_*x*_, *v*_*y*_, *v*_*z*_). Furthermore, the time dependence of a magnitude of the atom’s velocity (speed)  was a subject of further studies.

The LAMMPS input files, which explain how we modeled the silicon crystal phases, can be found by following the link in the Supporting Information.

### Ordinal Pattern Method

The atomic speed trajectory *v*(*t*_*i*_)|_*i*=0_^*N*–1^ was transformed into a trajectory of ordinal patterns π(*t*_*i*_)|_*i*=0_^*N*–1^, where *N* is the speed trajectory length, *m* defines the speed sequence length, *d* is the time lag, and *N*′ = *N* – (*m* – 1)*d* is the ordinal patterns trajectory length. Speed sequences
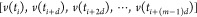
1contained in the speed trajectory were sorted in ascending order. In case of equal speed values, *v*(*t*) preceded *v*(*t*′) if *t* < *t*′. Then, an ordinal pattern (permutation σ of the *m*-element set) was assigned to each sorted speed sequence. Ordinal patterns were arranged lexicographically {σ_0_, ···, σ_*m*!–1_} to provide an integer representing σ. For example, if *m* = 4, *d* = 1, and *v*(*t*_*i*+1_) < *v*(*t*_*i*_) < *v*(*t*_*i*+3_) < *v*(*t*_*i*+2_)), then the speed sequence [*v*(*t*_*i*_), *v*(*t*_*i*+1_), *v*(*t*_*i*+2_), *v*(*t*_*i*+3_)] is represented by the ordinal pattern σ_7_ = (1032) and, therefore, π(*t*_*i*_) = 7.

The next step in the analysis of the atomic speed trajectory is to determine frequencies *f*_*i*_|_*i*=0_^*m*!–1^ of the ordinal pattern occurrence in the time series π(*t*_*i*_)|_*i*=0_^*N*–1^. As it will turn out, the frequency distribution obtained in this way allows distinguishing the crystalline phases of the tested material. However, from the practical point of view, it would require storing *m*! numbers per atom. A simplification can be achieved by calculating the entropy, normalized by the factor log_2_(*m*!):
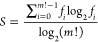
2

Thus, the idea of our approach is to assign a single number, *S*, to the atomic speed trajectory. We propose to use the name “S-method”, for simplicity.

**Figure 1:**
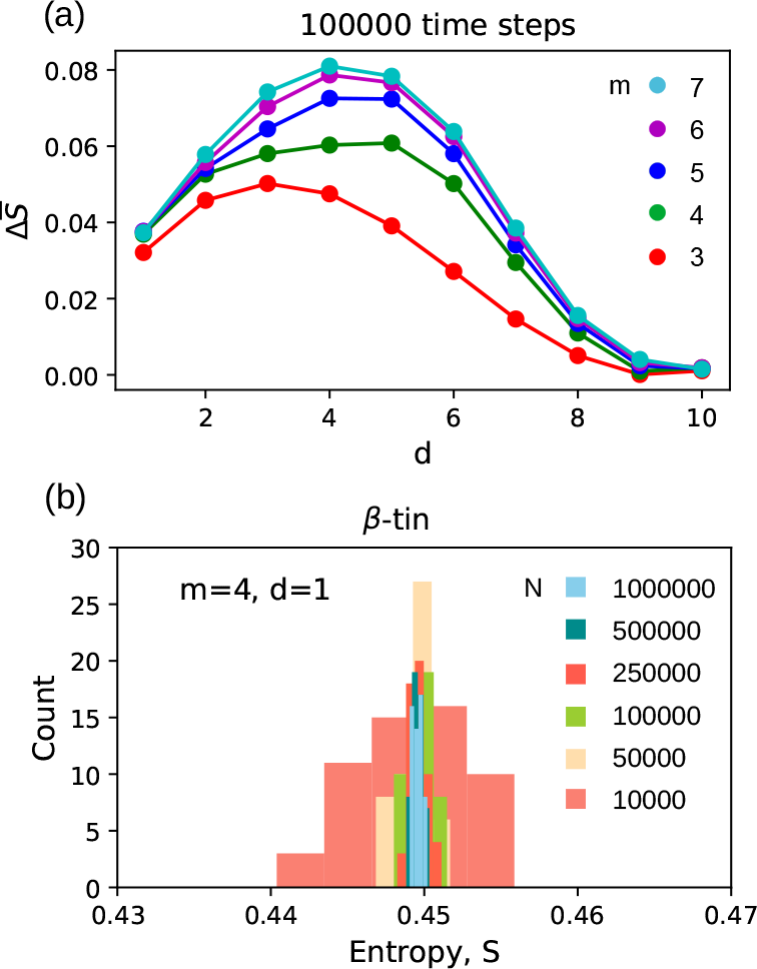
Case of β-tin/bct5 phases demonstrates the S-method’s primary properties. (a) Dependence of Δ*S̅* on the ordinal pattern length *m*, and the lag *d* calculated for the trajectory of *N* = 10^5^ time steps length. (b) Increase in the trajectory length caused a decrease in the entropy histogram width.

**Figure 2:**
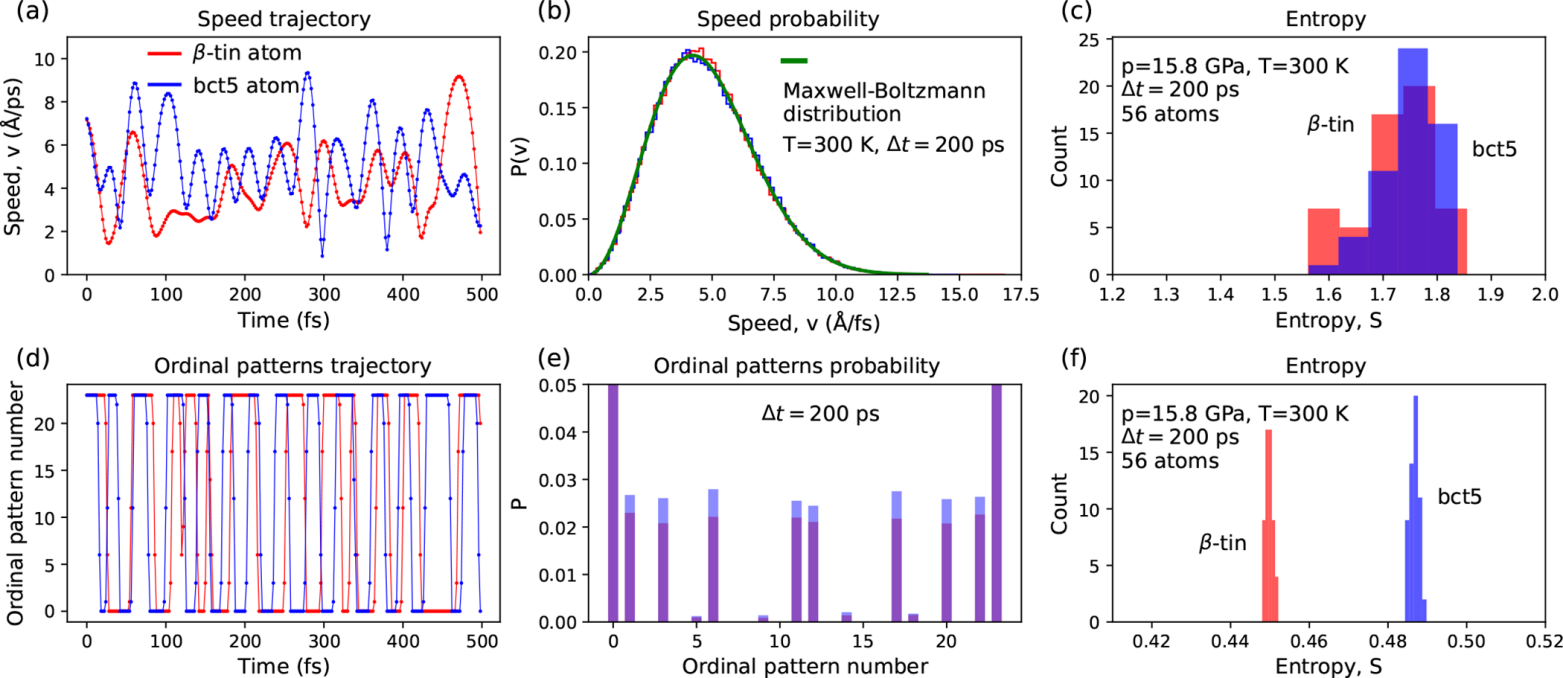
Method for distinguishing crystal structures based on speed dynamics. Left and middle columns present the speed trajectories of randomly selected one β-tin atom and one bct5 atom. Right column refers to the 56 atoms (not nearest neighbors) selected from each of the β-tin and bct5 crystals. (a) Time dependence of single-atom speed. Short time frame Δ*t* = 500 fs was chosen to present the trajectory details. (b) Probability distributions of speeds, *P*(*v*), calculated for the 200 ps trajectories and the theoretical Maxwell distribution. (c) Entropy of the probability distribution of speeds does not allow a clear distinction between β-tin and bct5 structures. (d) Symbolic representation of the single-atom speed trajectories in terms of the ordinal patterns. (e) Probability distribution of ordinal patterns, *P*. (f) Permutation entropy computed for the ordinal pattern probability distributions of β-tin and bct5 phase differ.

There are three parameters (*m*, *d*, *N*), which affect a value of the entropy. To recognize this relationship, we tested all pairs of Si phases indicated in Table 1. The finite length of the atomic trajectory pushed the study of the mean value *S̅* and standard deviation σ of the entropy dispersed over 56 atoms (not nearest neighbors) selected from each of the considered phases. The selected atoms were approximately evenly distributed throughout the crystal volume. Too many atoms could generate extremely large trajectory files. Certainly, we were curious how the phase separation Δ*S̅* = | *S̅*_phase1_ – *S̅*_phase2_| and mean standard deviation  depends on three natural numbers (*m*, *d*, *N*). We choose 3 ≤ *m* ≤ 7, 1 ≤ *d* ≤ 10, and 10^4^ ≤ *N* ≤ 10^6^ for tests as these intervals allowed investigation of essential properties of Δ*S̅* and σ̅. As the results of the analysis were similar, we decided to present the case of β-tin/bct5 (Figure 1). The results of the remaining tests can be found in the Supporting Information. Conclusions are as follows: (1) an increase of lag *d* caused an increase in Δ*S̅;* however, staring from a certain *d* value (depending on *m*), the trend is opposite (Figure 1a); (2) the value of σ̅ is at least an order of magnitude smaller than Δ*S̅*; and (3) an increase of the time series length *N* decreased a width (in fact the standard deviation) of the entropy histogram (Figure 1b).

Interestingly, Δ*S̅* ≈ 0 for lags *d* = 9,10 corresponded with the mean entropy approaching 1 (Figure S5a in the Supporting Information). For *m* = 2, there are two order patterns occurring with the frequency *f* ≈ 0.5, giving *S* ≈ 1, and consequently, the ordinal pattern length of *m* = 2 does not allow for distinguishing the phases (Figure S5b in the Supporting Information). We did not study the case of *m* > 7 due to the excessive number (*m*!) of order patterns.

For further investigation of silicon crystals, we chose *d* = 1 because it allows full utilization of the recorded trajectory. We chose *m* = 4 because it provides the smallest number of ordinal patterns and and exihibits larger Δ*S*_overlined, with respect to *m* = 3. Finally, we chose *N* = 10^5^. However, each of the tested values of *N* (from 10^4^ to 10^6^) allowed separation of the histograms of the studied phases (refer to the Supporting Information).

All data necessary to replicate the results of the performed simulations are provided in the Supporting Information.

## Results and Discussion

### Demonstration of the S-Method

We chose the β-tin/bct5 pair of Si phases to show how the S-method, distinguishing crystal structures, works (Figure 2). The results for other pairs of silicon phases can be found in the Supporting Information. As mentioned earlier, we investigated *N* = 10^5^ long atomic trajectories (200 ps); however, to compare the trajectories constructed from speeds and ordinal patterns, shorter 500 fs samples were used (Figure 2a,d).

As expected, the speed probability distributions *P*(*v*) calculated for two atomic trajectories (one β-tin atom and one bct5 atom) were consistent with the theoretical Maxwell–Boltzmann distribution (Figure 2b). Minor deviations caused by the finite length of the trajectory might give the illusion that the silicon phases can be distinguished by using *P*(*v*) or the corresponding entropy. To examine this presumption, we inspected the dispersion of the entropy value over groups of 56 atoms selected from both the β-tin and bct5 phases. The obtained histograms overlap (Figure 2c), indicating that the statistical analysis of the single-atom speed trajectory will not allow determination of whether an atom belongs to the β-tin or bct5 phase.

Then, we transformed the speed trajectory into the trajectory of the ordinal patterns (Figure 2d). The frequencies of the ordinal pattern occurrence differ (Figure 2e), exhibiting the ability to distinguish which phase the atom belongs. However, it requires comparing two frequency distributions, each composed of *m*! numbers. It is impractical, especially when we apply the S-method for tracking structural changes in a system containing hundreds of thousands or even millions of atoms. Instead of the frequency distribution of ordinal patterns, one can use a single number, namely, the entropy value. Figure 2f presents a comparison of two entropy histograms calculated for an equal number (56) of atoms of the btin and bct5 phases. The histograms are separated and have a small width.

Consequently, we showed that the statistical analysis of the single-atom ordinal pattern trajectory can determine the affiliation of an atom to a crystalline phase. Our method has given satisfactory results since it does not deal with a set of speeds but with a set of time-ordered short sequences of successive speeds. In the first case, every speed is treated individually and the deterministic relation connecting speed values is broken, while in the second case, this relation is partially preserved and affects the entropy value.

### Application to Other Silicon Phases

**Figure 3:**
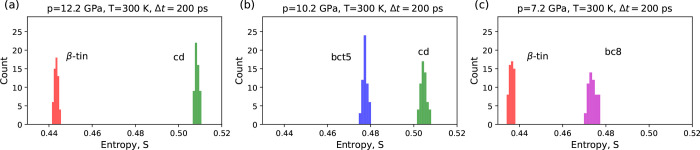
Distinguishing the silicon crystals modeled by Kumagai potential at *T* = 300 K and different equilibrium pressures (see Table 1). Histograms were computed for 56 atoms randomly selected from each of the cd (green), β-tin (red), bct5 (blue), and bc8 (magenta) crystals. Figures depict the relationship between the permutation entropy distributions and the high-pressure silicon phases: β-tin/cd (a), bct5/cd (b), and β-tin/bc8 (c). Separation of the entropy histograms confirmed the effectiveness of the S-method in investigation of the local atomic environment.

The relationship between atomic speed dynamics and crystal structure was established for the remaining silicon pairs: cd/bct5, cd/β-tin, and *beta*-tin/bc8 (Figure 3). As before, the entropy was calculated for 200 ps trajectories of 56 atoms selected from each of the examined phases. As before, the S-method allowed for the distinction of silicon structures. Notably, an increase in pressure shifts the entropy histograms toward higher values (compare the data presented in Figures 2f and 3). The qualitative explanation of this observation is based on the common perception of the entropy as a measure of information complexity. The increase in pressure reduces the interatomic distances, thus enhancing the influence of the atom’s surroundings on its movements. As a result, the atom’s speed trajectory becomes more complex, and the permutation entropy is shifted toward higher values.

### Comparison with Other Methods

The classical approach to studying the phase composition of the modeled system and the crystal lattice perturbations consists of analyzing data carrying information about the position of atoms at different stages of the simulation. The methods used for this purpose are often geometric, i.e., based on knowledge of interatomic distances and bond angles (the angle between the atom and its two closest neighbors).^1^ An important feature of these methods (e.g., centrosymmetry parameter (CS),^37^ coordination number analysis (CNA),^38^ and Voronoi tessellation) is assigning a particular property’s value to each atom by analysis of its local environment. Some methods examine the geometric relationships that exist within a group of atoms. For example, the radial distribution function (RDF) and the bond-angle distribution function (BADF) can be used to investigate the phase composition of a system since unique interatomic distances and bond angles characterize each crystal structure. However, due to thermal fluctuations in the positions of atoms, these unique values often blur, making phase identification challenging.

**Figure 4:**
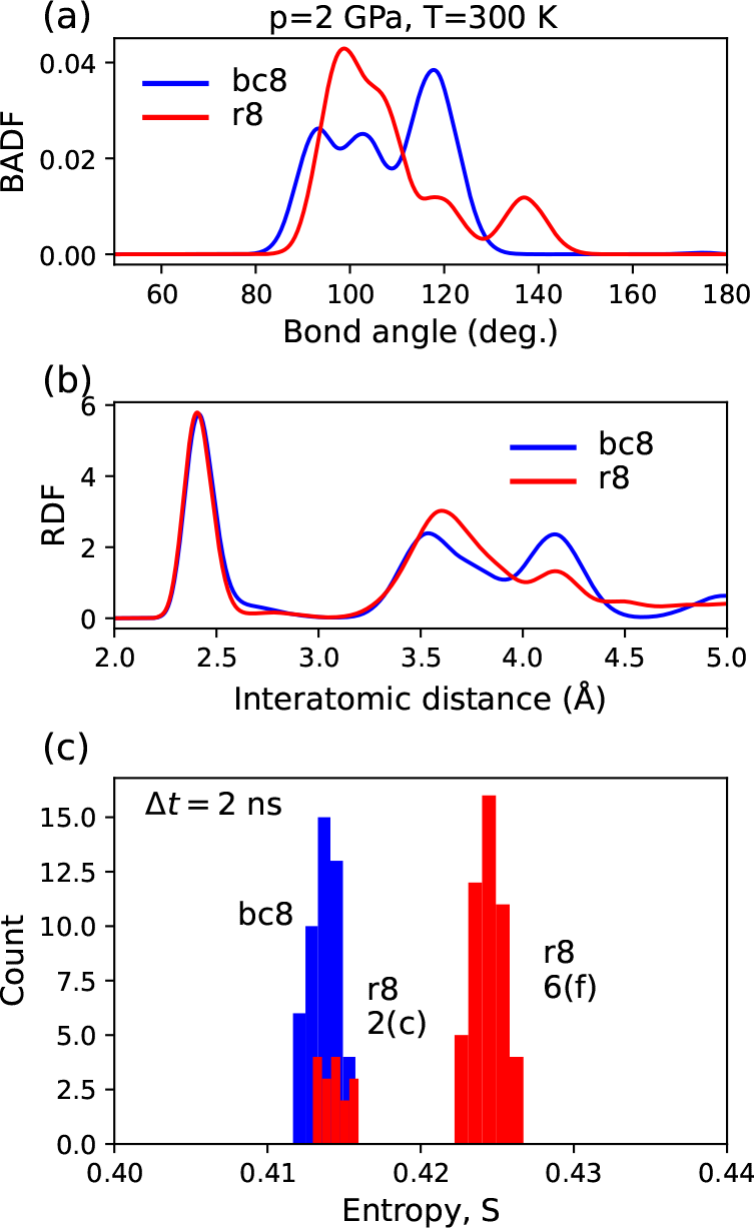
Discrimination of atoms that occupy different Wyckoff positions. Pertinent r8 and bc8 phases are modeled at a temperature of *T* = 300 K and a pressure of 2 GPa. (a) Distribution of bond lengths and (b) bond angles among nearest neighbors of the investigated r8 and bc8 atoms. (c) Application of the S-method to the trajectories of 2 ns length resulted in the separation of the permutation entropy distributions. This allows us to distinguish the atoms at the 2(c)-r8 (red) and 6(f)-r8 (red) Wyckoff positions. Separation of the histograms obtained for 6(f)-r8 and 16(c)-bc8 atoms (blue) allowed discernment r8 and bc8 crystals.

We will demonstrate this effect on the example of distinguishing bc8/r8 phases using RDF and BADF methods. Both crystals (each consisting of about 8000 atoms) were modeled at *T* = 300 K and *p* = 2 GPa using SNAP interatomic potential.^23,24^ We recorded 2 ns long trajectories of 64 r8 atoms and 48 bc8 atoms (not nearest neighbors). To calculate averaged interatomic distances and bond angles, we used the last 100 time steps of the crystals evolution. Figure 4a,b shows that the thermal motion of atoms causes the RDF and BADF peaks to broaden and consequently overlap. Thus, when studying (hypothetically) a mixture of two phases, one should expect RDF and BADF distributions with a shape similar to the sum of individual ones, which makes difficult determining whether the local maximum belongs to the bc8 or r8 phase. The ambiguity in the interpretation of the obtained results reflects the problem in experimental discerning between the r8 and bc8 phases.^17,18^ For example, the r8 phase participates in the pressure release-induced transformation from β-tin to bc8 phase in its final r8 → bc8 stage at *p* ≈ 2 GPa.^20^ In contrast, applying our S-method would allow the atoms to be divided into groups according to their entropy value. The atoms occupying the 2(c)-r8 and 16(c)-bc8 Wyckoff positions formed overlapped histograms. However, the entropy calculated for the r8 atoms occupying the 6(f) Wyckoff position was shifted toward higher values which made the differentiation of the r8 and bc8 structures successful. On this occasion, we showed that the S-method, focused on the statistical analysis of the atomic speed trajectory, distinguishes Wyckoff positions. Thermal motion, which blurs the RDF and BADF peaks and thus makes the identification of the r8 and bc8 phases difficult, is exploited by the S-method.

**Figure 5:**
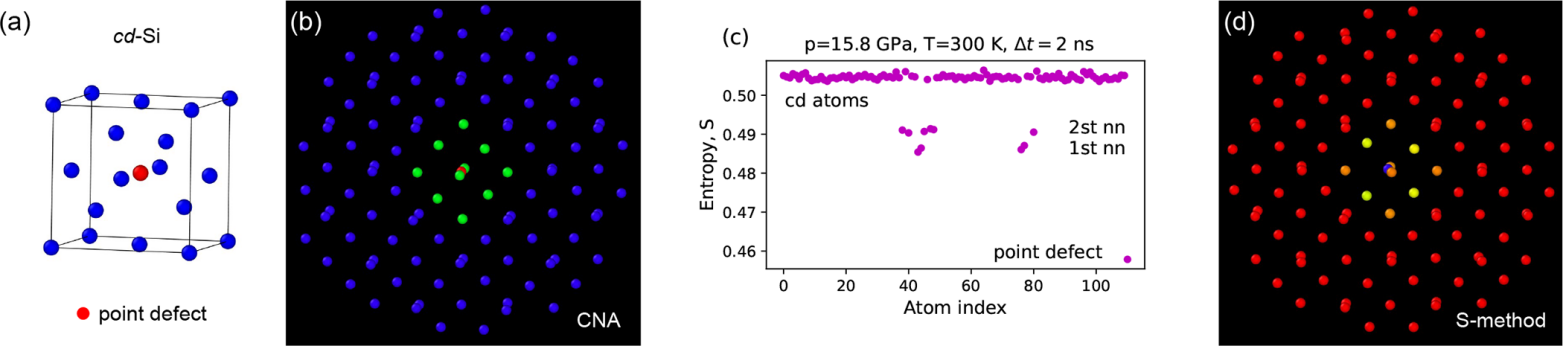
Analysis of local atomic environment of the Si self-interstitial defect. (a) Location of the point defect in the cubic diamond lattice of Si. (b) Result of examination using the common neighbor analysis (CNA) method. Coordination number for the point defect (red atom) and ten atoms (yellow color) of its environment equals 10 and 5, respectively. (c) Result of application of the S-method. Entropy of the point defect takes the lowest value. (d) Applying the S-method allowed for the distinguishing among the point defect (blue atom), first nn (yellow atoms), and 2th nn (orange atoms).

The next example of the S-method application concerns the Si self-interstitial defect.^39^ We considered a cubic diamond lattice and a silicon atom (Figure 5a) located at the (1/2,1/2,1/2) lattice site, i.e., in the center of the cube. In such a way, the tetrahedral defect was formed. Supercell containing the point defect was modeled in *T* = 300 K, *p* = 10.2 GPa, using the Kumagai potential.^22^ We recorded long trajectories (*N* = 10^6^ time steps) of the point defect and atoms that belong to its environment within a sphere of radius 10 Å. We were interested in the answer to the question whether the classical CNA method can distinguish point defect from the rest of atoms. We employed capabilities of the OVITO software.^40^ The CNA method assigns to each atom a coordination number *cn* equal to the number of neighbors located inside a sphere of a given radius *R*. Applying *R* = 3 Å, the CNA method localized the point defect (*cn* = 10, red atom in Figure 5b) and its 10 neighbors (*cn* = 5, green atoms); the coordination number of the rest silicon (blue) atoms was equal to 4. The S-method application allowed for more. Additionally, to indicating the location of point defect, it distinguished between the atoms of the closest surroundings of the point defect. Figure 5c shows the entropy values calculated for the atoms located within a sphere of radius 10 Å. The point defect has the lowest value *S* = 0.458 which distinguishes it from the four first nearest neighbors (nn) *S̅* = 0.486, six the 2st nn *S̅* = 0.491, and other atoms *S̅* > 0.5 (Figure 5d). The obtained result was not accidental, we tested the entropy values at temperatures from 200 to 600 K, and in each case, the hierarchy of the entropy values corresponded to the division of the system into a point defect, first nn, 2th nn, and the rest of the atoms, as shown in Figure 5c. Thus, by using the S-method, we localized the point defect and, moreover, distinguished the atoms in its vicinity.

## Conclusions

In summary, we showed that the speed trajectory of a single atom can be used to distinguish crystalline phases, including the discernible atoms occupying different Wyckoff positions. The proposed S-method is straightforward and can be applied to atoms without considering its surroundings. This feature contrasts geometrical analysis based on positional indicators of the atomic arrangement in the crystal lattice. Thermal fluctuations make it difficult to distinguish crystalline phases using geometrical methods but are, on the other hand, essential for the S-method. Based on the statistical analysis of ordinal patterns, our approach allows us to distinguish atoms belonging to different crystalline phases of silicon. This promising result indicates the possibility of using the S-method to study the structure and physicochemical properties of other materials modeled by molecular dynamics.

## Supporting Information Available

Dependence of enthalpy on pressure; S-method tests performed for β-tin/bct5, cd/β-tin, cd/bct5, and β-tin/bc8; and S-method applications (PDF)
