# Supplementary material for: Atom’s Dynamics and Crystal Structure: An Ordinal Pattern Method
Source: J Phys Chem A. 2025 Jan 17;129(4):1136–42. doi: 10.1021/acs.jpca.4c06151 (PMC11789147; doi:10.1021/acs.jpca.4c06151)
Supplement: Supplementary file 1 — jp4c06151_si_001.pdf [file jp4c06151_si_001.pdf]

Supplementary Information

Atom's dynamics and crystal structure: an ordinal pattern method

Rafal Abram[1], Roman Nowak[1,2] and Dariusz Chrobak[3]

[1] *Department of Chemistry and Materials Science, School of Chemical Engineering, Aalto University, Finland*
[2] *Institute of Scientific and Industrial Research, Osaka University, Osaka 567-0047, Japan*
[3] *Institute of Materials Engineering, University of Silesia in Katowice, Poland*

Corresponding author's email: dariusz.chrobak@us.edu.pl

The following link leads to LAMMPS input files, simulation results and Python codes (jupyter notebook) allowing to repeat all the results presented in the article.

Below, in points 1-6 we present the complete results obtained for the phases modeled with the Kumagai interatomic potential, as well as the results of the S-method tests.

1. The dependence of enthalpy on pressure, determined for the tested phases.

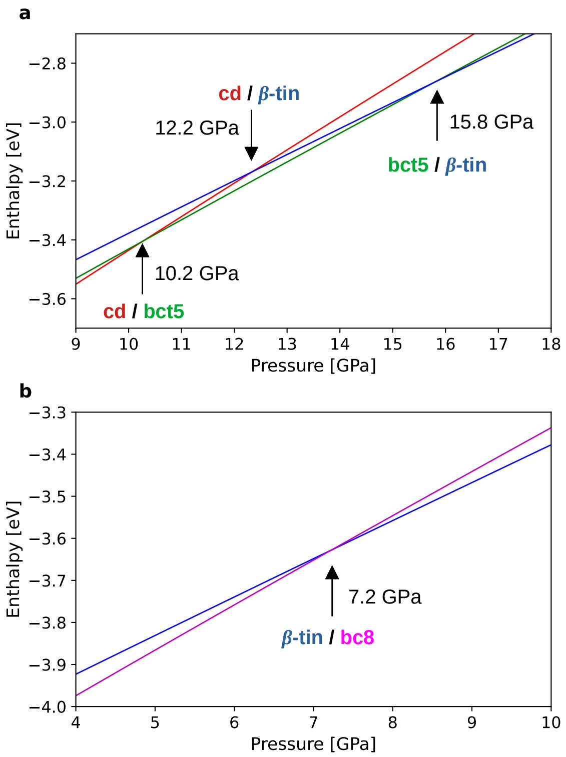

Fig. S1 The enthalpy (per atom) of high-pressure silicon phases versus pressure modeled with the Kumagai interatomic potential. The intersection point of the curves indicated the equilibrium pressure of the corresponding phases.

2. S-method tests performed for β-tin/bct5 (15.8 GPa) silicon phases.

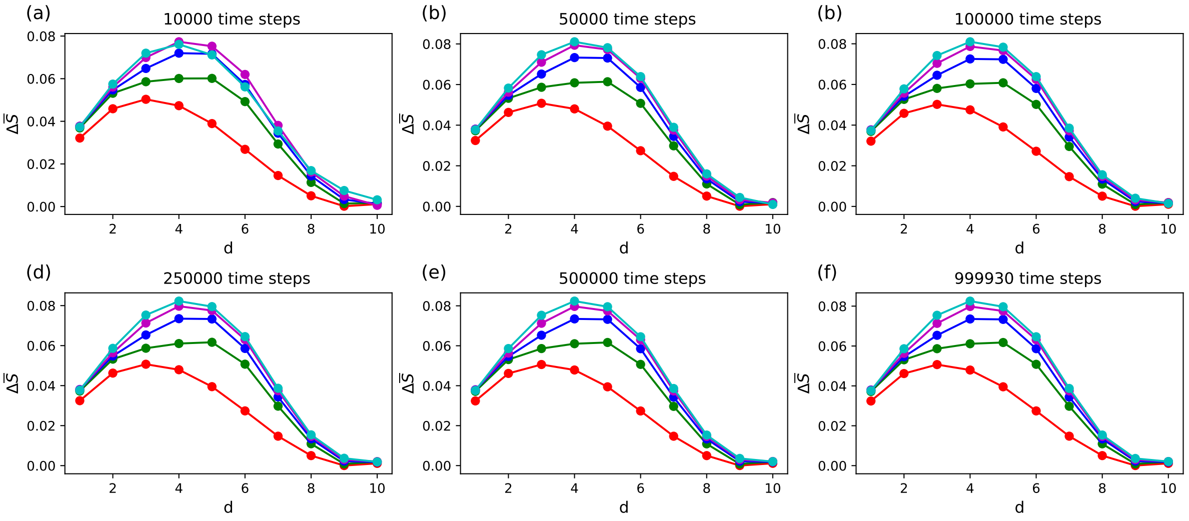

Fig. S2 Dependence of $\Delta\overline{S}$ on the numbers ($m.d,N$), calculated for β-tin/bct5 system. Refer to Fig. 1a for the color meaning.

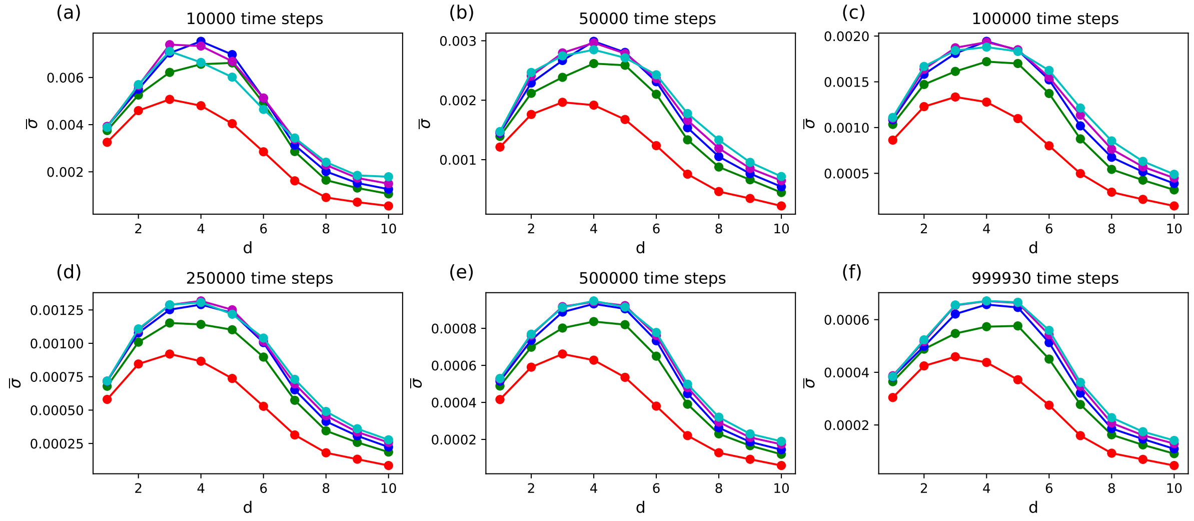

Fig. S3 Dependence of $\overline{\sigma}$ on the numbers ($m.d,N$), calculated for β-tin/bct5 system. Refer to Fig. 1a for the color meaning.

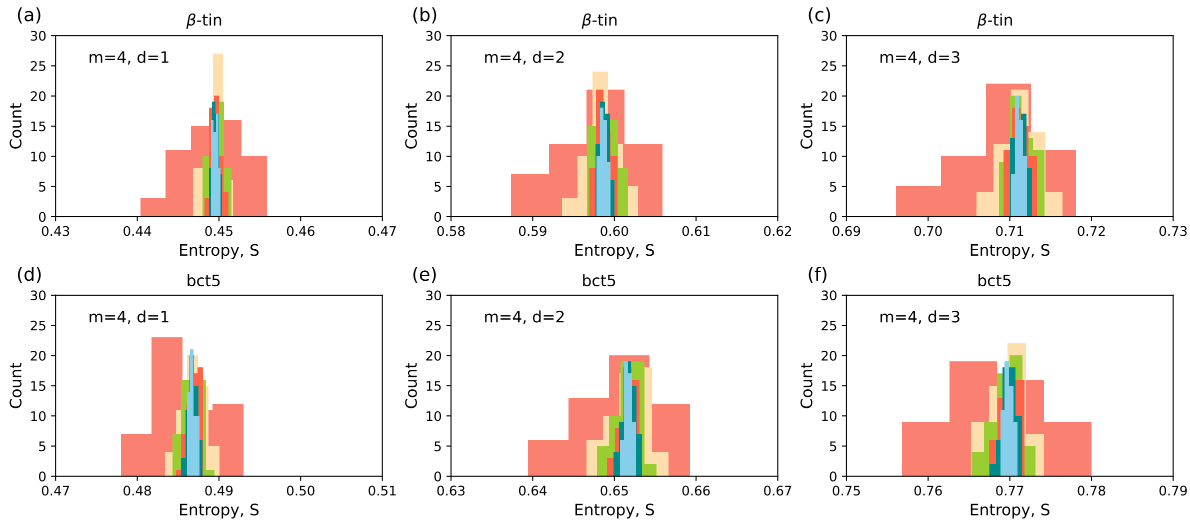

Fig. S4 Presentation of the entropy histograms (m=4, d=1,2,3) showing a decrease of $\overline{\sigma}$ with respect to N. Refer to Fig. 1b for the color meaning.

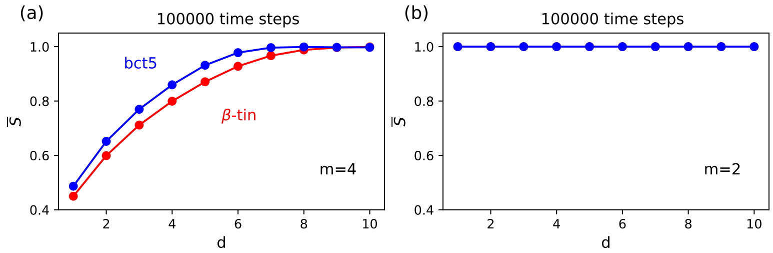

Fig. S5 (a) Increasing the lag *d* value caused the entropy to approach 1, as shown for *m*=4 and *N*=10^5 time steps. (b) Very short order patterns (*m*=2) did not allow for distinguishing the crystals at all.

3. S-method tests performed for cd/β-tin (12.2 GPa) silicon phases.

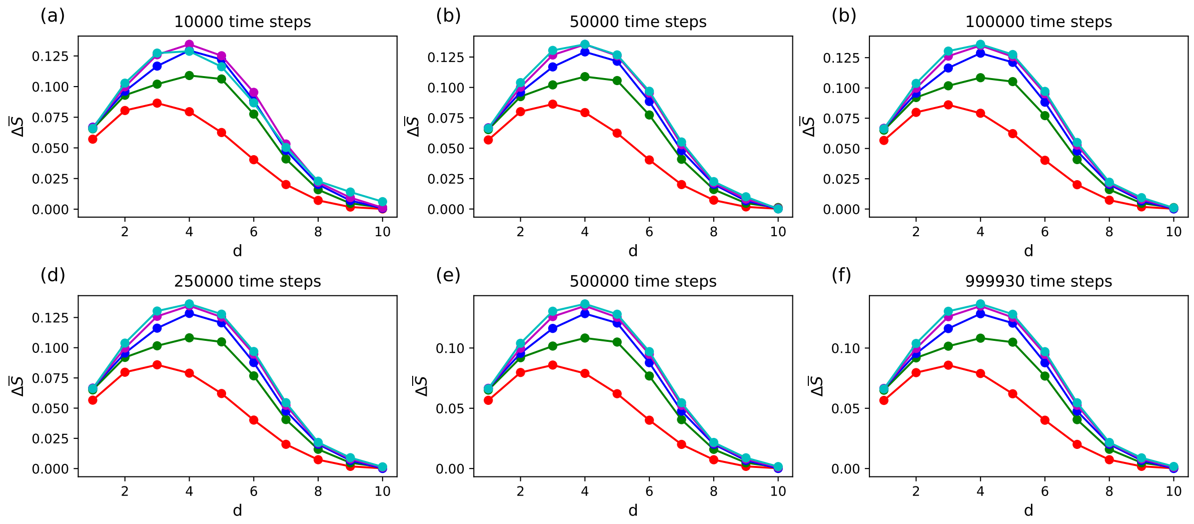

Fig. S6 Dependence of $\Delta\overline{S}$ on the numbers ($m.d,N$), calculated for cd/β-tin system. Refer to Fig. 1a for the color meaning.

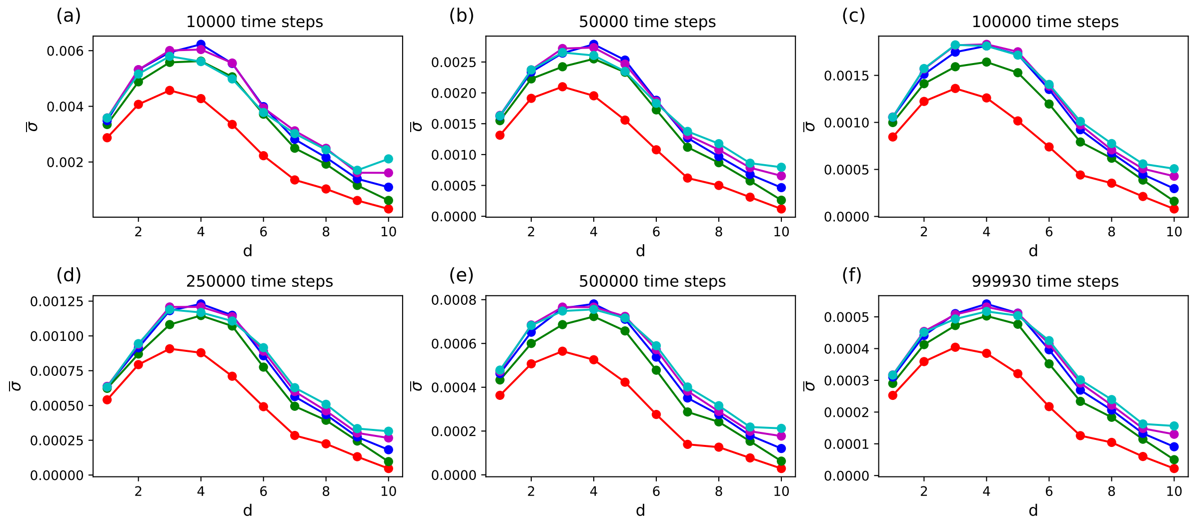

Fig. S7 Dependence of $\overline{\sigma}$ on the numbers ($m.d,N$), calculated for cd/β-tin system. Refer to Fig. 1a for the color meaning.

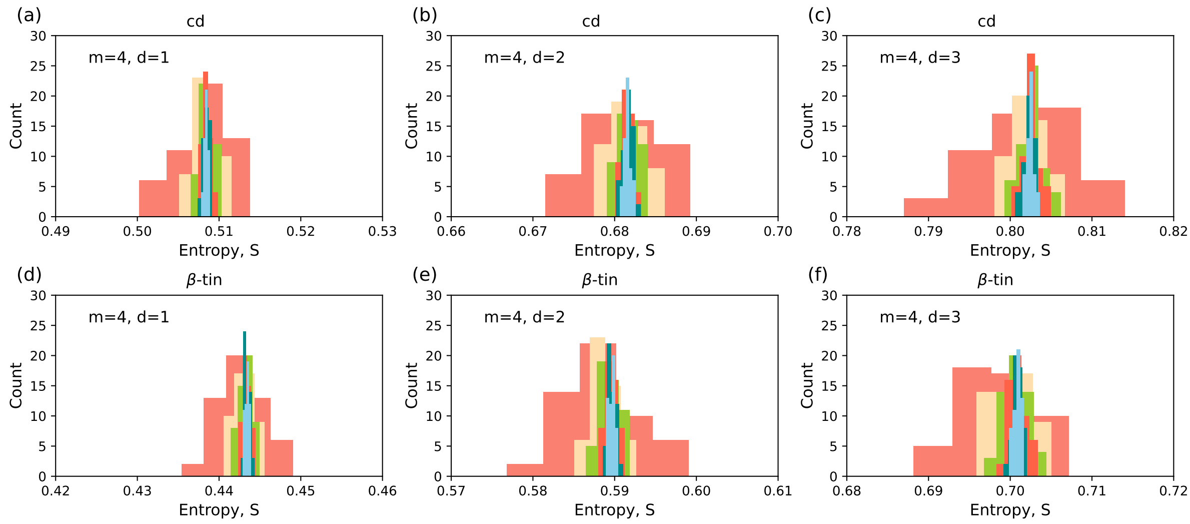

Fig. S8 Presentation of the entropy histograms (m=4, d=1,2,3) showing a decrease of $\overline{\sigma}$ with respect to N. Refer to Fig. 1b for the color meaning.

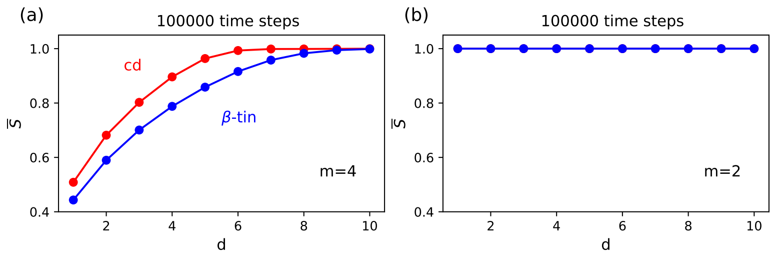

Fig. S9 (a) Increasing the lag *d* value caused the entropy to approach 1, as shown for *m*=4 and $N=10^5$ time steps. (b) Very short order patterns (*m*=2) did not allow for distinguishing the crystals at all.

4. S-method tests performed for cd/bct5 (10.2 GPa) silicon phases.

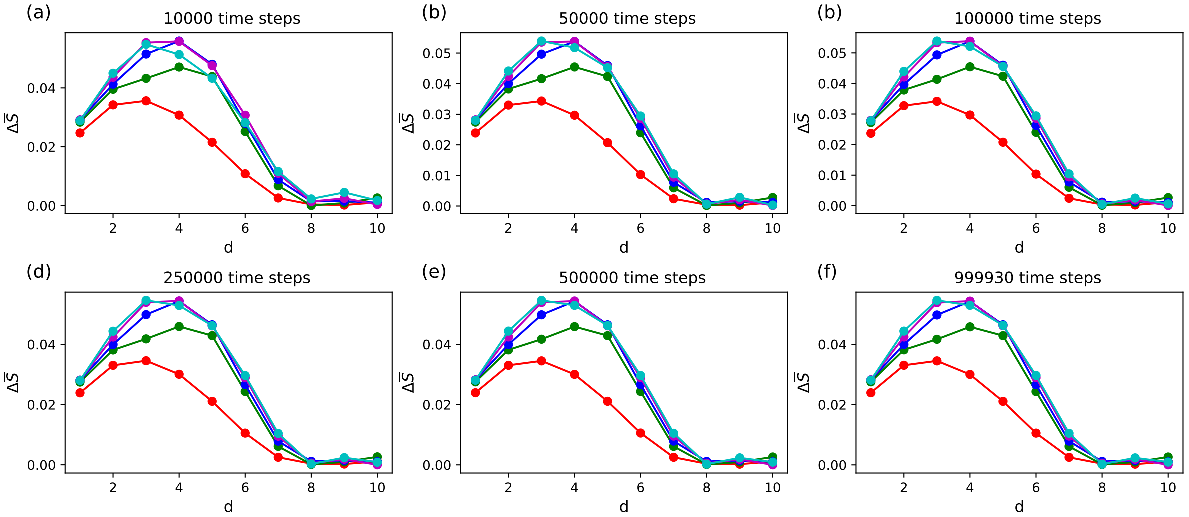

Fig. S10 Dependence of $\Delta\overline{S}$ on the numbers (*m.d,N*), calculated for cd/bct5 system. Refer to Fig. 1a for the color meaning.

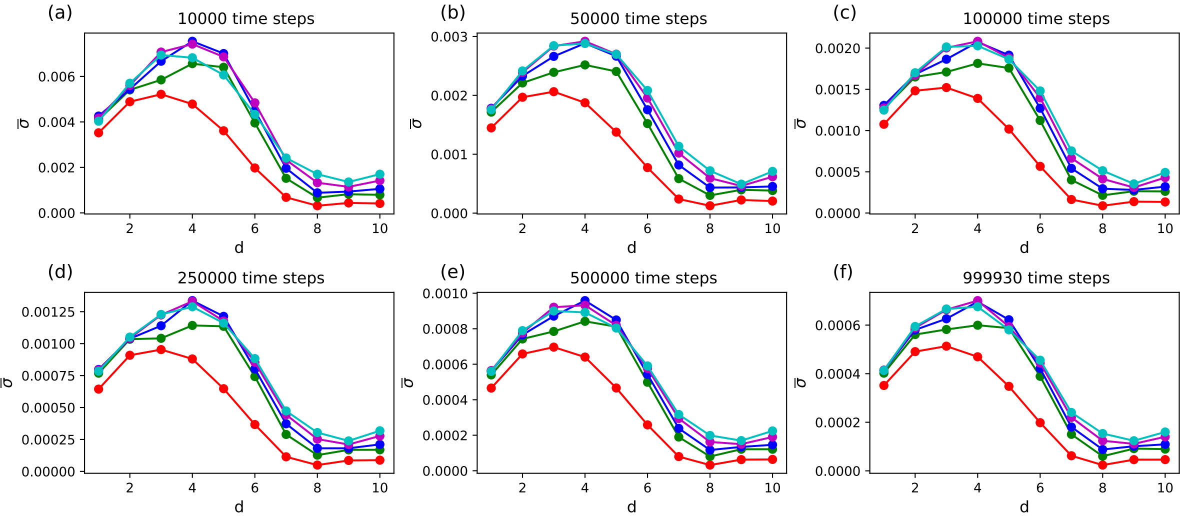

Fig. S11 Dependence of $\overline{\sigma}$ on the numbers (*m.d,N*), calculated for cd/bct5 system. Refer to Fig. 1a for the color meaning.

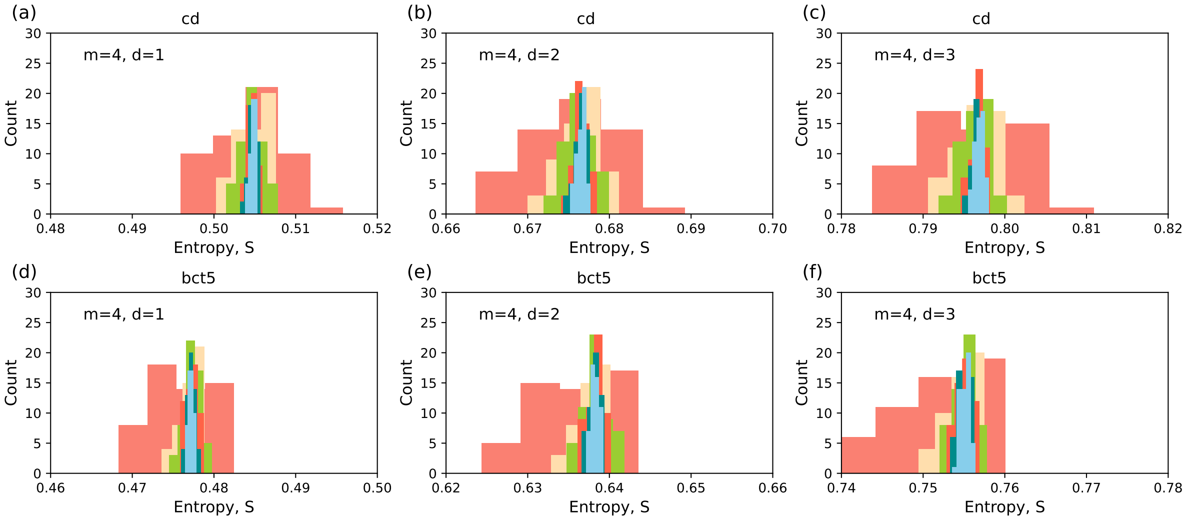

Fig. S12 Presentation of the entropy histograms (*m*=4, *d*=1,2,3) showing a decrease of $\overline{\sigma}$ with respect to *N*. Refer to Fig. 1b for the color meaning.

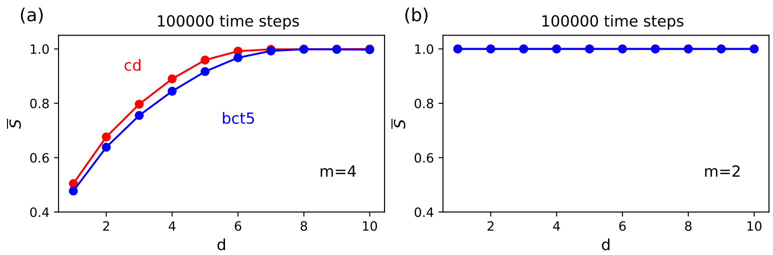

Fig. S13 (a) Increasing the lag *d* value caused the entropy to approach 1, as shown for *m*=4 and *N*=10^5 time steps. (b) Very short order patterns (*m*=2) did not allow for distinguishing the crystals at all.

5. S-method tests performed for β-tin/bc8 (7.2 GPa) silicon phases.

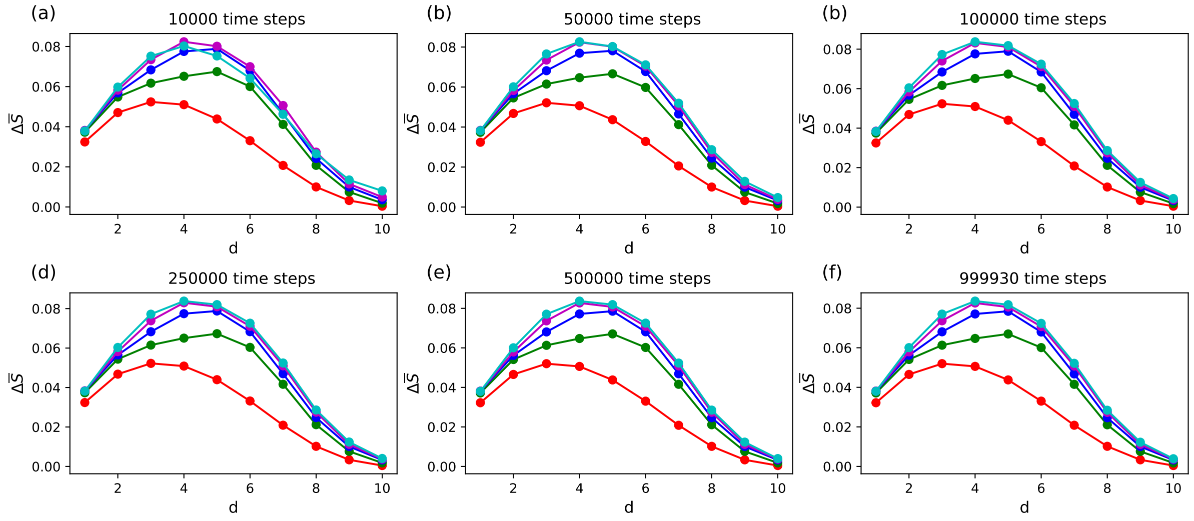

Fig. S14 Dependence of $\Delta\overline{S}$ on the numbers ($m.d,N$), calculated for β-tin/bc8 system. Refer to Fig. 1a for the color meaning.

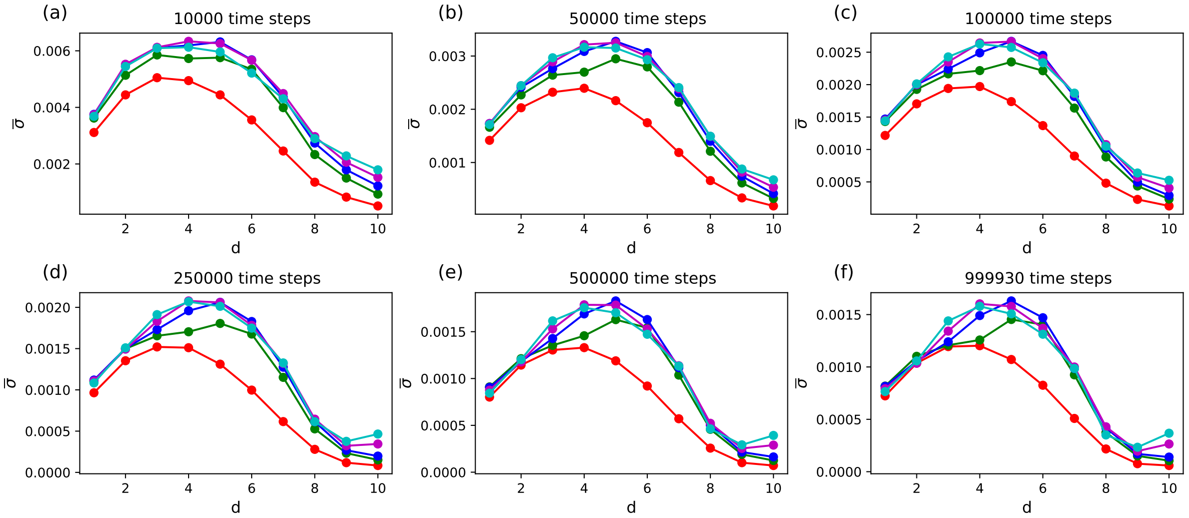

Fig. S15 Dependence of $\overline{\sigma}$ on the numbers ($m.d,N$), calculated for β-tin/bc8 system. Refer to Fig. 1a for the color meaning.

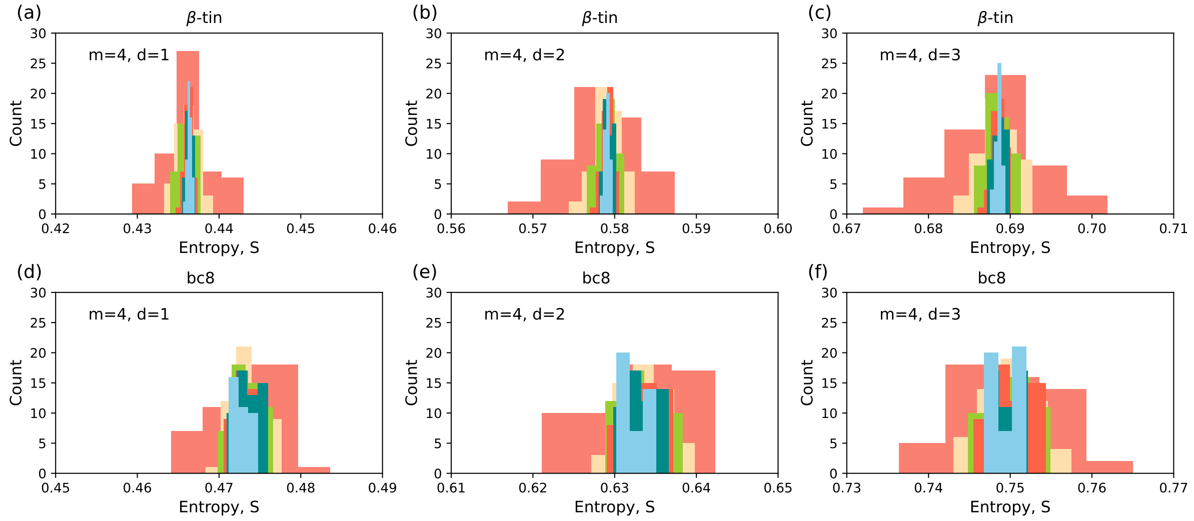

Fig. S16 Presentation of the entropy histograms (m=4, d=1,2,3) showing a decrease of $\overline{\sigma}$ with respect to N. Refer to Fig. 1b for the color meaning.

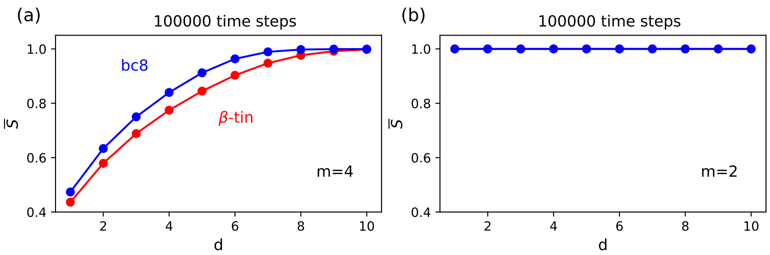

Fig. S17 (a) Increasing the lag d value caused the entropy to approach 1, as shown for m=4 and N=10^5 time steps. (b) Very short order patterns (m=2) did not allow for distinguishing the crystals at all.

6. S-method applications.

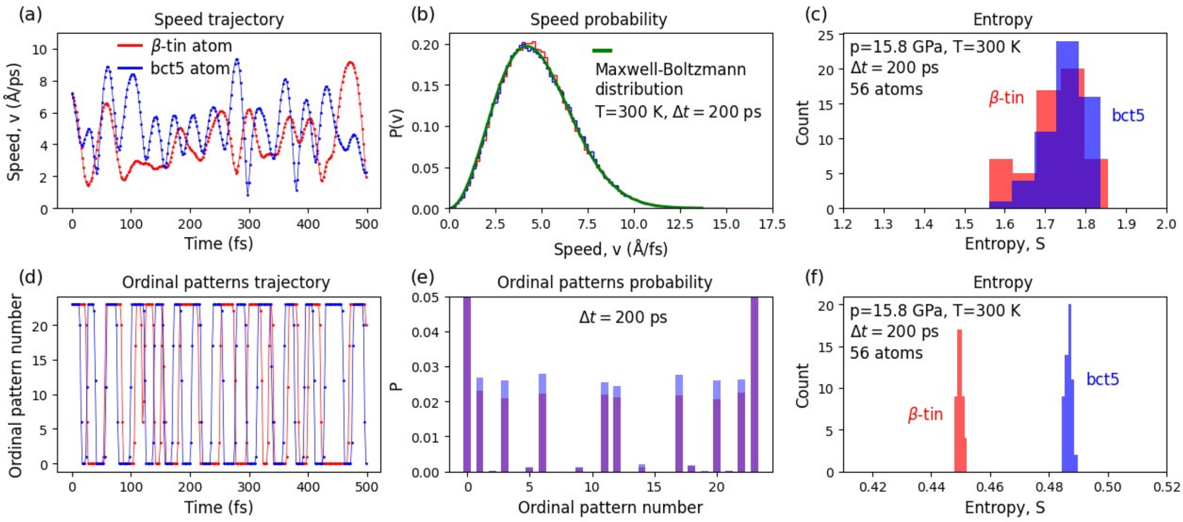

Fig. S18 A method for distinguishing crystal structures (β-tin/bct5) based on speed dynamics.

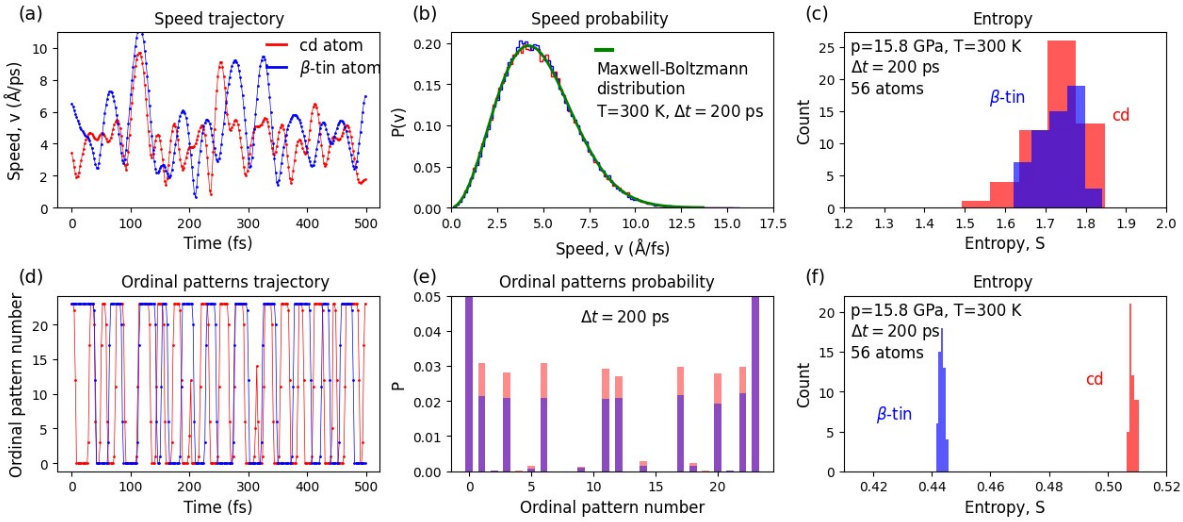

Fig. S19 A method for distinguishing crystal structures (cd/β-tin) based on speed dynamics.

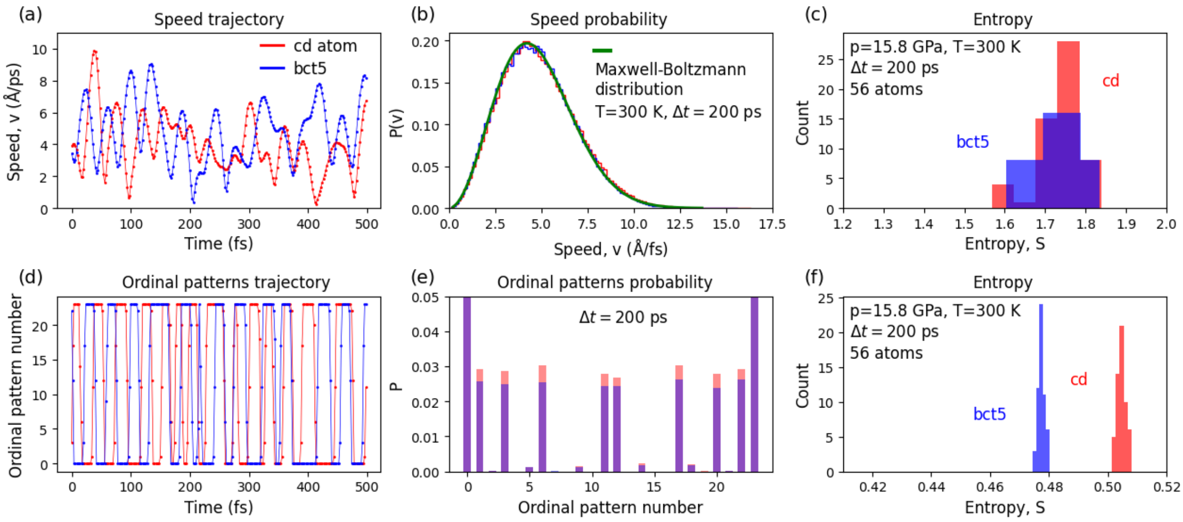

Fig. S20 A method for distinguishing crystal structures (cd/bct5) based on speed dynamics.

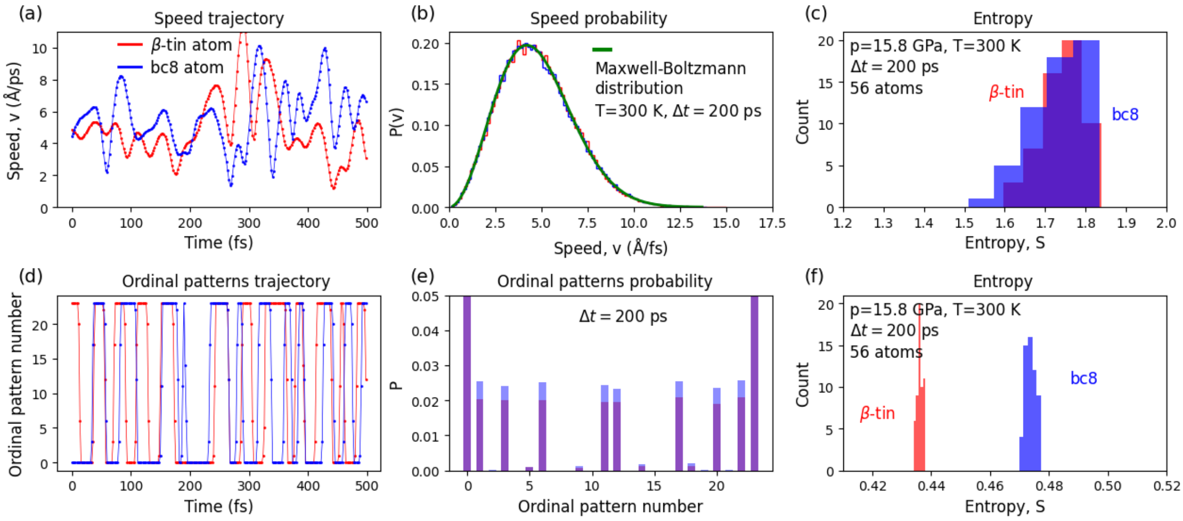

Fig. S21 A method for distinguishing crystal structures (β-tin/bc8) based on speed dynamics.
